# Contrasting pathogen prevalence between tick and dog populations at Chornobyl

**DOI:** 10.1186/s13071-024-06563-4

**Published:** 2024-11-17

**Authors:** Megan N. Dillon, Barbara A. Qurollo, Rachael Thomas, Madeline E. Warren, Timothy A. Mousseau, Jennifer A. Betz, Norman J. Kleiman, Matthew Breen

**Affiliations:** 1 Department of Molecular Biomedical Sciences, College of Veterinary Medicine, North Carolina State University, Raleigh, NC USA; 2 Department of Clinical Sciences, College of Veterinary Medicine, North Carolina State University, Raleigh, NC USA; 3 Department of Biological Sciences, North Carolina State University, Raleigh, NC USA; 4 Department of Biological Sciences, University of South Carolina, Columbia, SC USA; 5 Clean Futures Fund, Godfrey, IL USA; 6 Visiting Veterinarians International, Damascus, OR USA; 7 Department of Environmental Health Sciences, Mailman School of Public Health, Columbia University, New York, NY USA; 8 Comparative Medicine Institute, North Carolina State University, Raleigh, NC USA; 9 Center for Human Health and the Environment, North Carolina State University, Raleigh, NC USA; 10 Cancer Genetics, UNC Lineberger Comprehensive Cancer Center, University of North Carolina, Chapel Hill, NC USA; 11 Duke Cancer Institute, Duke University, Durham, NC USA

**Keywords:** Chernobyl, Chornobyl, *Ixodes ricinus*, Ticks, Tick-borne pathogens, Zoonotic pathogens, Free-breeding dogs, Quantitative PCR, Droplet digital PCR

## Abstract

**Background:**

The 1986 disaster at the Chornobyl Nuclear Power Plant released massive amounts of radioactive material into the local environment. In addition to radiation, remediation efforts and abandonment of military-industrial complexes contributed to contamination with heavy metals, organics, pesticides and other toxic chemicals. Numerous studies have evaluated the effects of this contamination on the local ecology. However, few studies have reported the effect of this contamination on vector-borne pathogens and their hosts. In this manuscript, we characterize tick-borne pathogen presence at two sample locations within the Chornobyl Exclusion Zone, one at the Nuclear Power Plant (NPP) and another 16 km away in Chornobyl City (CC).

**Methods:**

Ticks and whole-blood samples were collected from free-breeding dogs captured at the NPP and CC. Endpoint PCR and quantitative PCR were used to identify tick species and to assess the presence of specific tick-borne pathogens, including *Anaplasma phagocytophilum*, *Borrelia burgdorferi* sensu lato, *Babesia* spp., *Bartonella* spp., *Francisella tularensis* and general *Anaplasmataceae*. A droplet digital PCR assay was developed for *Babesia canis* and *A. phagocytophilum* to evaluate their presence in dogs from the two populations. Pathogen prevalences between the two sample populations were compared by calculating *Z*-scores.

**Results:**

Ticks were identified as *Ixodes ricinus* (*n* = 102) and *Dermacentor reticulatus* (*n* = 4). Overall, 56.9% of *I. ricinus* ticks were positive for at least one pathogen. A significantly higher prevalence of *A. phagocytophilum* and *B. burgdorferi* was found in ticks at the NPP (44.0% and 42.0%, respectively) compared to CC (23.1% and 19.2%, respectively). *Babesia* spp*.* (including *B. canis* and *B. caballi*) were detected in 8.8% ticks at similar proportions for both populations. Interestingly, we found a significantly lower level of *A. phagocytophilum* in dogs at the NPP (1.8%) than in dogs at CC (11.7%). In total, 24.3% of dogs were positive for *B. canis*, evenly distributed across the two populations.

**Conclusions:**

The results of this study show contrasting pathogen prevalence in both ticks and dogs at the NPP and CC, which may reflect the differential exposures at the two locations. This work adds an important new component to our understanding of the consequences of prolonged exposure to environmental contamination on the wildlife and ecology within the Chornobyl Exclusion Zone.

**Graphical Abstract:**

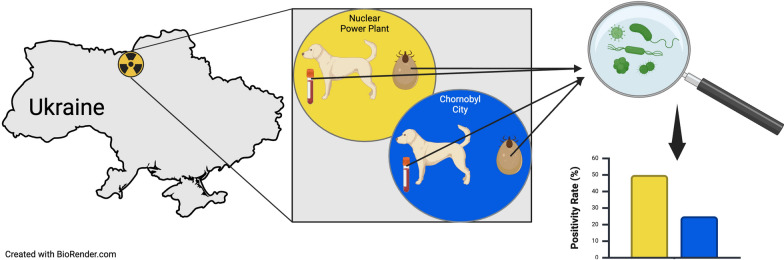

**Supplementary Information:**

The online version contains supplementary material available at 10.1186/s13071-024-06563-4.

## Background

In 1986, the steam explosion and meltdown that destroyed reactor 4 at the Chornobyl Nuclear Power Plant (NPP) complex caused massive radionuclide contamination of the surrounding territories of Ukraine, Belarus and Russia. More than 5000 petabecquerels (PBq) of radionuclides were released by the explosion and subsequent fires, including large quantities of long-lived ^137^Cs and ^90^Sr radioisotopes, whose adverse environmental effect will persist for greater than eight half-lives, about 240 years [1]. Radionuclide contamination, however, is only one of many serious adverse environmental effects resulting from the disaster and the decades-long cleanup efforts that followed. These include widespread contamination by heavy metals, organics, pesticides, asbestos and other pollutants introduced by remediation efforts, as well as the abandonment of an industrial complex containing, in addition to the four operating nuclear power plants, power substations, industrial facilities, a military base and massive construction projects, including a new cooling tower and two more nuclear power plants [2–6]. The large numbers of diverse contaminants make the environment surrounding the Chornobyl NPP unique. Nevertheless, in the absence of human habitation, and despite the hazardous conditions, many wildlife species now have established populations within the roughly 4000 km^2^ Chornobyl Exclusion Zone (CEZ) [7]. The re-emergence of flora and fauna within the CEZ has provided a unique opportunity for scientists to study the multi-generational effects of exposure to the contaminants at Chornobyl and has been the focus of numerous research studies.

Specifically, it is important to understand and characterize the potential health risks to human populations that continue to work or visit within the CEZ and to the resident animal and plant species. Over the past decades, animal studies in the CEZ have highlighted decreased population abundances (e.g. [8]), severe impacts on fertility of certain species (e.g. [9]), ocular pathologies (e.g. [10]) and morphological changes (e.g. [11]), while work in other species fails to note any significant effects in exposed populations (e.g. [7, 12–15]). In addition to population-level assessments, other scientists have investigated vector-borne pathogen levels in the areas just outside of the Chornobyl NPP in the CEZ [16–18]. Two of these studies reported that the prevalence of certain pathogens is different at sites closer to the NPP when compared to regions outside of the CEZ [16, 18]. Understanding the prevalence of pathogens in local vectors is another way to assess health risks in both animal and human populations arising from the disaster.

Studies that have focused on hard ticks within the CEZ highlighted increased levels of certain pathogens, such as *Anaplasma phagocytophilum* and *Babesia canis* in *Dermacentor reticulatus* ticks along with *Borrelia burgdorferi* sensu lato (*B. burgdorferi* s.l.) and *Bartonella* spp. in *Ixodes ricinus* ticks [16–18]. Increased prevalence of pathogens within the tick populations across the region could result in greater spread of diseases in the wild populations upon which the ticks feed. The increased presence of *B. canis*, *B. burgdorferi* and *A. phagocytophilum* are specifically of interest, as the respective diseases these organisms cause can be fatal in some species when left untreated [19–21]. Other authors have focused directly on the effect of radiation and other contaminants on infectious diseases and their vectors [22–27]. Exposure to radioisotopes was reported to reduce infectivity and survival of ticks based on the level of exposure and, in some cases, affected the diversity, prevalence and pathogenicity of pathogens (reviewed in [28]). Other studies also reported that small mammals, which can serve as reservoir hosts for different tick-borne pathogens, had elevated levels of pathogens and vectors in areas affected by radioactive contamination (reviewed in [28]). These authors noted that effects in reservoir species and vectors can have great impacts on the exposure of host species to vector-borne pathogens, including accidental hosts like companion animals and people. In addition to the importance of understanding how the Chornobyl disaster may have impacted pathogens and the risks they may pose to the local communities, it has been noted that the recent Russian invasion into Ukraine could facilitate the spread of vector-borne pathogens into other regions as animals are relocated [29]. It is, therefore, critical to understand the prevalence of these pathogens in the tick populations within the CEZ to better assess possible health effects for the resident populations and to understand how this may affect surrounding regions.

Along with the numerous wildlife species that have re-established in this area, several hundred free-breeding dogs now inhabit the CEZ, descendants of household pets that survived the extreme contamination and concerted extermination efforts [6, 30]. In previous studies, we found high levels of genetic differentiation and low levels of migration between two geographically close populations of these dogs: one population at the NPP and one 16 km away in Chornobyl City (CC) [31, 32]. As an expansion of our previous work, in the study reported here we used samples from these two distinct populations of dogs, characterized by their proximity to the NPP, to better understand the impact of radiation on vector-borne pathogen levels. We addressed this by determining the species and level of pathogens detected in ticks and their canine hosts when sampled at the NPP and further away at CC. We hypothesized that there are different prevalences of pathogens in ticks and their canine hosts when sampled at the NPP as compared to the less contaminated site in CC.

## Methods

### Sample collection

Ticks were collected from free-breeding dogs during spay/neuter/vaccination efforts conducted at the NPP and CC sample sites in 2018 sponsored by the Clean Futures Fund (described in [31, 32]). Collected ticks were immediately preserved in 70% ethanol on site until processed. We acquired a total of 106 ticks from 36 different dogs that were captured either at the NPP (*n* = 17 dogs; 54 ticks) or in CC (*n* = 19 dogs; 52 ticks). The majority (78%) of the ticks were visibly engorged upon collection and the remainder (22%) were flat. After removal from 70% ethanol, each tick was weighed and photographed prior to DNA extraction. All of the collected ticks were morphologically assessed for life-stage identification. Since the majority of ticks were engorged, tick species identification was conducted via molecular methods. Six of the sampled ticks were collected while mating to the feeding tick, but these males were separated out before DNA extraction.

In a previous study we also previously acquired blood-derived DNA samples, taken in 2019 from 111 unique dogs, of which 55 were sampled at the NPP and 56 sampled in CC (detailed further in [31]), along with DNA samples taken in 2018 for 24 dogs (20 at NPP and 4 at CC) that were sampled again in 2019. To maintain continuity between sampling years and provide equal representation for both populations, we focused on the 2019 samples for assessment of pathogen prevalence. We also determined pathogen presence in the 2018 samples, but these samples were used primarily to assess persistence and not for overall population prevalence. The ticks collected from dogs in 2018 overlapped with five of these dogs for which we have a blood sample from 2018. The 2019 dog samples used in the pathogen prevalence analysis did not have corresponding tick samples in the present analysis.

### DNA extraction

**Fig. 1:**
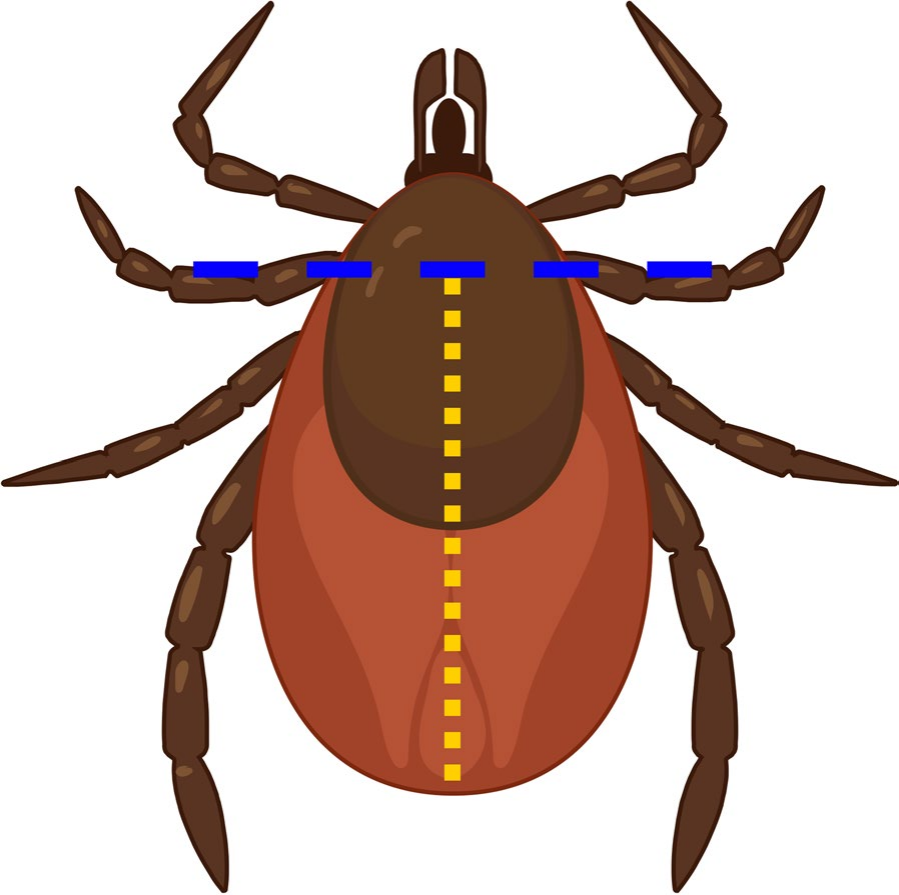
Schematic of tick dissection prior to DNA extraction, where DNA was extracted from one half of the body and from the front-most portion of the scutum. Created with BioRender (https://www.biorender.com/)

Prior to extraction, we dissected each tick with a sterilized scalpel, removing the mouthparts and the front-most part of the scutum (hereafter, ‘head’) and bilaterally dissecting the ‘body’ (Fig. 1). We then extracted DNA from each ‘head’ and each half of the bisected ‘body’ so that we could make an attempt to differentiate which pathogens were only present in the blood meal, i.e. only in ‘body’ extracts, from those that may be in the salivary glands, which could be in both ‘head’ and ‘body’ extracts. DNA was extracted from the ticks following the protocol of the Qiagen Blood and Tissue DNA extraction kit (Qiagen, Hilden, Germany) with a few alterations. We increased the digestion time, allowing the tissue to incubate at 56 °C in buffer ATL and proteinase K overnight, together with the inclusion of additional proteinase K after 12 h, vortexing intermittently. For the largest engorged ticks, a second dose of buffer ATL and proteinase K was added after the initial 12-h incubation and the incubation time was extended for another 12 h. DNA was eluted twice with same 60 µL of high-performance liquid chromatography water. DNA integrity and purity was assessed by agarose gel electrophoresis and spectrophotometry (NanoDrop One spectrophotometer; Thermo Fisher Scientific, Waltham, MA, USA). All extracts were stored at - 20 °C prior to analysis.

### Tick species identification

For species identification, DNA isolated from each tick ‘head’ was used as the template for PCR amplification of an 820-bp region of the mitochondrial cytochrome* c* oxidase subunit I gene (*COI*) (all primer sequences are included in Table 1) [33]. The PCRs were performed in reaction volumes of 20 µl total, containing 20 ng of template DNA, 1× Taq RED Master Mix (Apex Bioresearch), and 300 nM of each primer. PCR assays were performed following the protocol described in Lv et al. [33]. Amplicons were sequenced using conventional bidirectional Sanger sequencing by the North Carolina State University Genomic Sciences Laboratory (NCSU GSL). Each sequenced amplicon was analyzed using National Center for Biotechnology Information (NCBI) BLAST to determine species identity, based on the lowest e-value of the matching NCBI sequence that shared > 95% identity with the amplicon.

**Table 1:** Primers and probes used in PCR assays

Endpoint PCR primers			
Assay target	Primer name	Sequence (5′–3′)	References
Tick COI (820 bp)	Cox1-F	GGAACAATATATTTAATTTTTGG	Lv et al. [33]
	Cox1-R	ATCTATCCCTACTGTAAATATATG	
*Anaplasmataceae* (257 bp)	AnaSpp-F	GGGGATGATGTCAARTCAGCAY	Krücken et al. [35]
	AnaSpp-R	CACCAGCTTCGAGTTAAGCCAAT	
*Francisella tularensis* (357 bp)	FrancTul-F	GCAGGTTTAGCGAGCTGTTCTACTC	Kormilitsyna et al. [36]
	FrancTul-R	AGCTGTCCACTTACCGCTACAGAAG	
*Borrelia spp.* (210 bp)	Borr16s-F	AGTGGCGAACGGGTGAGTA	Qurollo et al. [38]
	Borr16s-R	CTCTCAGGCCGGTTACTTATC	
Quantitative PCR primers and probes			
Assay target	Primer name	Sequence (5'-3')	References
*Ixodes ricinus* (probe-based qPCR; 130 bp)	IxRic-F	CTGGAGCTTCCGTTGACATAG	Robinson et al. [34]
	IxRic-R	GGTATTCGTTCTAAAGATAGTCCTGGT	
	IxRic-P-HEX	TCCCTTCATTTAGCAGGAATTTCATCA	
*Anaplasma phagocytophilum* (probe-based qPCR/ddPCR; 76 bp)	ApMSP2-F	TGGAAGGTAGTGTTGGTTATGGTATT	Courtney et al. [37]
	ApMSP2-R	TTGGTCTTGAAGCGCTCGTA	
	ApMSP2-FAM	TGGTGCCAGGGTTGAGCTTGAGATTG	
*Borrelia burgdorferi* (probe-based qPCR; 77 bp)	Bb23s-F	CGAGTCTTAAAAGGGCGATTTAGT	Courtney et al. [37]
	Bb23s-R	GCTTCAGCCTGGCCATAAATAG	
	Bb23s-P-FAM	AGATGTGGTAGACCCGAAGCCGAGTG	
*Rickettsia raoultii* (probe-based qPCR; 107 bp)	RrOmpB-F	GTGGTGGTGTTCCTAATACTCC	Jiang et al. [39]
	RrOmpB-R	ACCTAAGTTGTTATAGTCTGTAGTAAAC	
	RrOmpB-P-FAM	TATTGGCACTGTACAGTTAAAGCA	
*Bartonella spp.* (probe-based qPCR; 253 bp)	BssrA-F	GCTATGGTAATAAATGGACAATGAAATAA	Diaz et al. [40]
	BssrA-R	GCTTCTGTTGCCAGGTG	
	BssrA-P-FAM	ACCCCGCTTAAACCTGCGACG	
*Babesia spp.* (SYBR qPCR w/ melt Curve; 150 bp)	B-lsu-F	ACCTGTCAARTTCCTTCACTAAMTT	Qurollo et al. [41]
	Bmic-F	TTGCGATAGTAATAGATTTACTGC	
	B-lsu-R	TCTTAACCCAACTCACGTACCA	
*Piroplasmid* (SYBR qPCR w/ melt curve; 200 bp)	Piro18s-F	GCAGTTAAAAAGCTCGTAGTTGAATT	Tyrrell et al. [42]
	Piro18s-R	GTTAAATACGAATGCCCCCAA	
*Babesia canis* (ddPCR; 88 bp)	Bcanis-F	TAGTTTGAAACCCGCCTT	Kuo et al. [43]
	Bcanis-R	GATGGGTCAGAAACTTGAA	
	Bcanis-P-HEX	CATCGCTAAATGCGATTCGCCA	
*Canis lupus familiaris* STXBP6 (ddPCR; 104 bp)	STXBP6-F	CCAGGATTCTGCAGAGTTTGAT	Mochizuki et al. [44]
	STXBP6-R	GTGGTGGAGGATTTGGAAGAAG	
	STXBP6-P-HEX	AATGCCTTTGACCAGTGGGTAGCC	

*COI* mitochondrial cytochrome* c* oxidase subunit I,* dd PCR* droplet digital PCR,* F* forward* qPCR* quantitative PCR,* R* reverse

We additionally used a quantitative PCR (qPCR) assay for the detection and quantification of *I. ricinus* DNA, which was performed as described in Robinson et al. [34]. Although the primers and hydrolysis probe (IxRic) were designed to detect *I. ricinus*, initial testing indicated that the assay was also able to detect *D. reticulatus* DNA at a higher cycle threshold. This served as a confirmation for the COI species identification method. We tested the reaction on the three different cycle conditions described for each pathogen probe-based qPCR. The IxRic assay then served as an internal positive control (IPC) in all four of the hydrolysis probe-based qPCRs to verify that each test DNA sample was PCR-competent. All qPCR assays were conducted using a CFX384 Touch Real-Time PCR System (Bio-Rad Laboratories, Hercules, CA, USA).

### PCR detection of pathogens

Two traditional endpoint PCRs were used to detect tick-borne pathogens, one for *Anaplasmataceae* spp. and one for *Francisella tularensis*. For both PCRs, we used the undiluted ‘head’ DNA extract as template, as the ‘body’ DNA extracts contained high concentrations of DNA from both tick and host. Using the ‘head’ DNA extracts allowed for more accurate detection of pathogens that were present in the tick and not simply present in the blood meal.

The *Anaplasmataceae* PCR primers were used to detect different species within the *Anaplasmataceae* family [35]. Each 20-µl reaction volume contained 1× GoTaq G2 Master Mix (Promega, Madison, WI, USA), 300 nM of each forward and reverse primer, and 2 µl of template DNA. PCR was performed as described in Krücken et al. [35].

The PCR for *F. tularensis* was used to detect the presence of different subspecies of *F. tularensis* [36]. The final 20-µl reaction volume contained 1× GoTaq G2 Master Mix, 480 nM of each primer and 2 µl of template DNA. The PCR thermal cycling profile was as described in Kormilitsyna et al. [36]. All products, for both the *Anaplasmataceae* and the *F. tularensis* PCR were visualized in a 2% agarose gel in 1× TAE, and amplicons were bidirectionally sequenced at the NCSU GSL. NCBI BLAST was used to identify the origins of each amplicon.

### qPCR detection of pathogens

We conducted five qPCRs targeting pathogen species or species complexes that had been documented previously as being detected in ticks located around the CEZ. This included hydrolysis probe-based qPCRs for *A. phagocytophilum* (ApMSP2), *B. burgdorferi* s.l. (Bb23s), *Rickettsia raoultii* (RrOmpB), and *Bartonella* spp. (BssrA), in addition to a SYBR qPCR for detection of *Babesia* spp. (Table 1). Each of the hydrolysis probe-based qPCRs designed to detect pathogens was duplexed with the IxRic assay, which served as an IPC to ensure amplification competency in all reactions. As qPCR allows for increased sensitivity and accuracy, we first analyzed undiluted DNA extracts from the ‘body’ of the ticks, despite the higher DNA concentrations. We then followed up by testing each corresponding ‘head’ DNA extract for each ‘body’ sample that was PCR positive. All samples, including unknowns and controls (positive/negative/no-template), were run in duplicate. Presence of pathogen was indicated when both duplicate unknown samples produced an amplicon of the expected size for matched ‘body’ and ‘head’ extracts, as determined by agarose gel electrophoresis.

We used species-specific primers and a hydrolysis probe targeting the *MSP2* gene for the detection and quantification of *A. phagocytophilum* [37]. The final 25-µl reaction volume contained 1× PerfeCTa qPCR ToughMix (QuantaBio, Beverly, MA, USA), 2 µl of template DNA, 900 nM of each AsMSP2 primer, 125 nM of AsMSP2 probe and 250 nM of IxRic primers and probe. PCR for both ApMSP2 and Bb23s were performed following the protocol described in Courtney et al. [37]. Two dog-derived blood samples positive for *A. phagocytophilum* were used as positive controls for this analysis, and these samples were acquired from the NCSU Vector Borne Disease Diagnostic Laboratory (VBDDL). We used a synthetic control, at a range of 10^6^ copies to 10^1^, for standard curve calculations.

For the detection and quantification of *B. burgdorferi*, we used primer and a hydrolysis probe sequences targeting the *23S* gene [37]. The final 25-µl reaction volume contained 1× PerfeCTa qPCR ToughMix, 2 µl of template DNA, 700 nM of each Bb23s primer, 175 nM of Bb23s probe and 250 nM of IxRic primers and probe. We used a synthetic control designed for a 490-bp region surrounding the targeted amplicon as the positive control, diluted to 10^3^ copies. We used the synthetic control to calculate a standard curve for dilutions from 10^6^ to 10^1^. For tick samples yielding amplicons from this assay, we then assessed the genospecies using endpoint PCR and Sanger sequencing. Primers used for this assay targeted the* 16S* gene for *Borrelia* spp. [38]. Each 25-µl reaction volume contained 1× GoTaq G2 Master Mix, 600 nM of each Bor16S primer and 2 µl of template DNA. The PCR thermal cycling profile followed the protocol described in Qurollo et al. [38]. Products were visualized in a 2% agarose gel in 1× TAE. Amplicons were then sequenced bidirectionally at the NCSU GSL, and BLAST was used to type the genospecies of *B. burgdorferi* within the positive ticks.

For the detection and quantification of *R. raoultii*, we used primers and a probe that was modified from a molecular beacon to a hydrolysis probe to target the *OmpB* gene [39]. Each 25-µl reaction volume for RrOmpB and IxRic contained 1× PerfeCTa qPCR ToughMix, 2 µl of template DNA, 500 nM of each RrOmpB primer, 400 nM of RrOmpB probe and 250 nM of IxRic primers and probe. The PCR thermal cycling profile following that described in Jiang et al. [39]. We used a synthetic control designed for a 414-bp region surrounding the targeted amplicon as the positive control, diluted to 10^3^ copies. We also used the synthetic control to calculate a standard curve for dilutions from 10^6^ to 10^1^.

For cross-genus detection of *Bartonella*, we used primers and a probe targeting the *ssrA* gene [40]. This assay (BssrA) was designed for species identification upon sequencing of the amplicon. The final 25-µl reaction volume, duplexed with IxRic, contained 1× PerfeCTa qPCR ToughMix, 2 µl of template DNA, 500 nM of each BssrA primer, 400 nM of BssrA probe and 250 nM of IxRic primers and probe. The PCR thermal cycling profile followed the protocol described in Diaz et al. [40]. Two feline-derived samples that were known to harbor *Bartonella henselae* were used as positive controls for this analysis, which were acquired from the NCSU VBDDL.

For the detection of *Babesia* spp., we used a SYBR qPCR assay described in Qurollo et al. [41]. The 12.5-µl reaction volume contained 1× SsoAdvanced Universal SYBR Green Supermix (Bio-Rad Laboratories), 600 nM of B-LSU-F and B-LSU-R2, 400 nM of BMic-F and 2 µl of template DNA. PCR and melt curve analysis were performed as described in Qurollo et al. [41]. We looked specifically for melting temperatures between 76.5 °C and 77 °C for confirmation of *Babesia* spp. presence, and sequenced amplicons at the NCSU GSL to ensure specificity in amplification and to identify the *Babesia* species. Two canine-derived samples positive for *Babesia vogeli*, acquired from the NCSU VBDDL, were used as positive controls. Since this region shows high genetic similarity between *B. canis* and *B. vogeli*, we performed an additional SYBR qPCR to better identify these species of *B. canis* for ticks identified as positive through the aforementioned *Babesia* assay. This piroplasmid assay targets a 188-bp region of the* 18S* gene and has increased specificity towards *B. canis* over *B. vogeli* [42]. Each 25-µl reaction volume contained 1× SsoAdvanced Universal SYBR Green Supermix, 100 nM of each piroplasmid primer, and 2 µL of template DNA. The PCR and melt curve analysis were performed as described in Tyrrell et al. [42]. Resultant amplicons were sequenced bidirectionally at NCSU GSL and assessed with BLAST to further confirm the species identity of *Babesia* within positive ticks.

### Pathogen droplet digital PCR assay for dogs

Using droplet digital PCR (ddPCR), we assessed the presence of pathogens in DNA samples from the peripheral blood of dogs located at either the NPP or in CC. The use of ddPCR specifically allowed for greater sensitivity for detecting circulating pathogens in these blood samples. We focused on two pathogens prevalent in the tick populations that infect canine blood cells: *A. phagocytophilum* and *Babesia* spp. We first tested a random canine DNA sample for the presence of these two pathogens using the ApMSP2 and the *Babesia* spp*.* qPCR assays described above for pathogen detection in the ticks, and then designed a more sensitive ddPCR assay to test each of the canine DNA samples. The preliminary assessment indicated the presence of both of these pathogens within dogs from both populations, and further identified that the species of *Babesia* present was *B. canis* (via Sanger sequencing of the positive amplicons). Based on these results from the test samples, we adapted qPCR assays for the detection of *A. phagocytophilum* (FAM) and *B. canis* (HEX), as described in [43], to form a single duplexed two-color ddPCR assay, thereby providing increased sensitivity in our level of detection. We established the optimum annealing temperature of 60 °C for the duplexed ddPCR, and this ddPCR assay was then used to detect these two prominent pathogens in blood-derived DNA samples from dogs of both populations.

Initially, we assessed all 135 blood-derived Chornobyl DNA samples (111 unique dogs) and 100 DNA samples from healthy, uninfected dogs for amplification competency with a probe-based ddPCR assay targeting a region on dog chromosome 8 (described in [44]). To examine any evidence of spurious hydrolysis of the probe in the reaction mixture, we first tested the competent DNA isolates from the 100 uninfected dogs. We then tested all competent Chornobyl dog samples for the presence of *A. phagocytophilum* and *B. canis* with the duplexed ddPCR assay. We performed the analysis using the manufacturer’s recommended protocol for primer and probe concentrations (900 nM of primers, 250 nM of probe), 1× ddPCR Supermix for probes [no dUTP]; Bio-Rad Laboratories), 2 µl of undiluted DNA extract as template (equivalent to approx. 10 µl of whole dog blood) and water for a final reaction volume of 22 µl. Droplets were generated with an Automated Droplet Generator (Bio-Rad Laboratories). PCR thermal cycling was performed using a C1000 Thermal Cycler (Bio-Rad Laboratories), starting with an initial denaturation step of 95 °C for 10 min, followed by 40 cycles of 94 °C for 30 s and 60 °C for 60 s, with a final extension at 98 °C for 10 min. Droplets were then analyzed using a QX200 Droplet Reader (Bio-Rad Laboratories). Each analysis plate contained positive controls (synthetic DNA controls at a concentration of 10^3^ copies), no-template controls and negative controls (uninfected dog DNA). The positive controls were used to determine the expected amplitude for each channel.

### Statistical analysis

For each pathogen, we calculated a *Z*-score to compare the proportion of positivity between the two populations. We used the following equation to calculate the *Z*-scores, based on the null hypothesis that the difference between the proportion for each population is zero.

$${\rm Z}= \frac{\left({\widehat{p}}_{1}- {\widehat{p}}_{2}\right)-0}{\sqrt{\widehat{p}\left(1- \widehat{p}\right) \left(\frac{1}{{n}_{1}}+\frac{1}{{n}_{2}}\right)}}$$

where:

$$\widehat{p}= \frac{{Y}_{1}+{Y}_{2}}{{n}_{1}+ {n}_{2}}$$

A significant difference in proportions of population positivity, based on an alpha value of 0.05 and a two-tailed hypothesis, would require a *Z*-score with an absolute value > 2.

## Results

### Tick species identification

All 106 ticks were identified via* COI* amplification and sequencing; of these, 102 (93.6%) were *I. ricinus* and four (3.8%) were *D. reticulatus.* All four of the *D. reticulatus* ticks were sampled from the NPP population. Morphological assessment indicated that all collected ticks were adults.

All ticks amplified via the IxRic qPCR when tested in conjunction with each of the pathogen assays, despite the design targeting only *I. ricinus*. The four *D. reticulatus* ticks amplified at a higher cycle threshold (Ct) collectively than the *I. ricinus* (average Ct 32 vs 16, respectively). The consistent amplification confirmed the competency of the template DNA to amplify when duplexed with the pathogen qPCR assays.

### PCR detection of pathogens

**Fig. 2:**
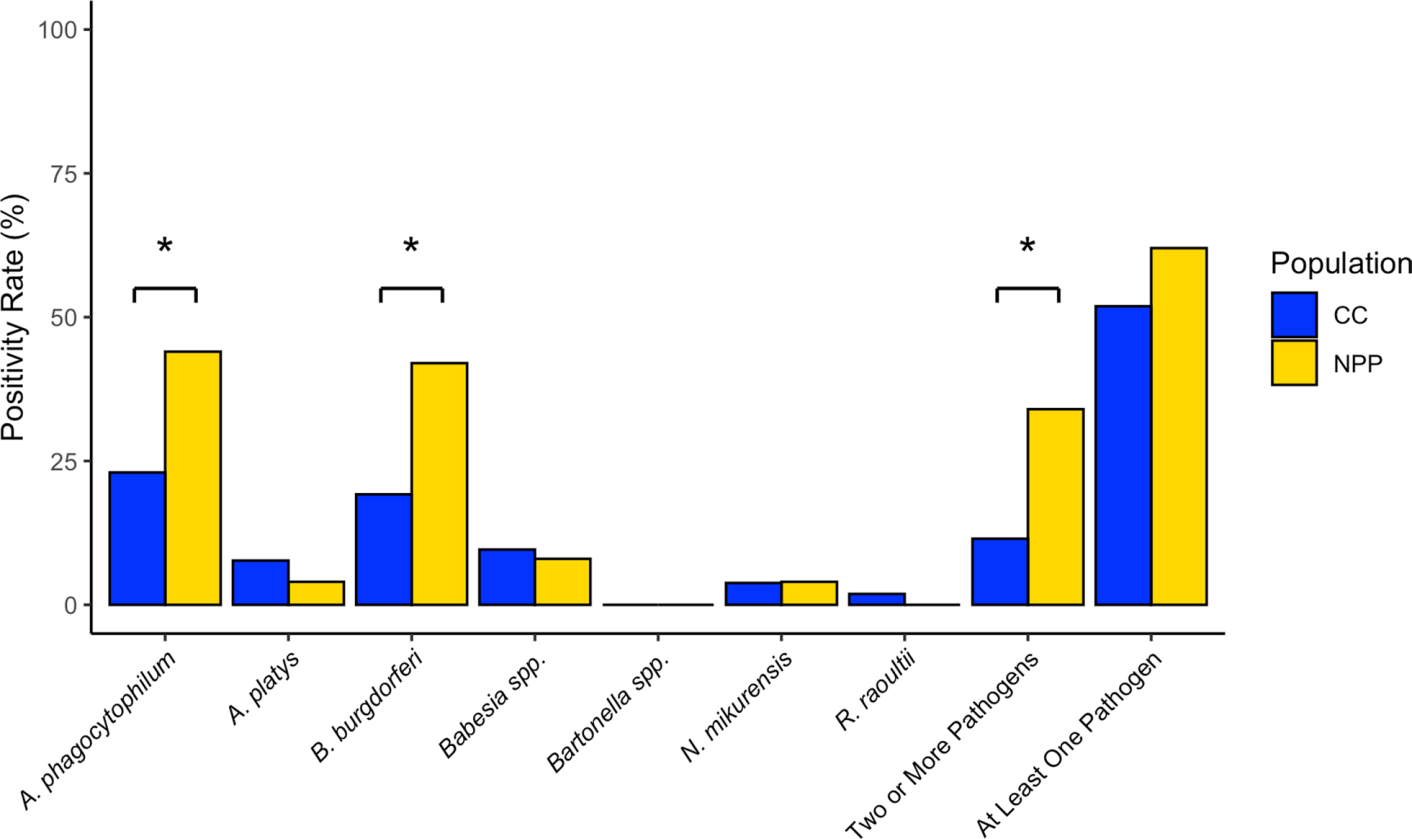
Pathogen prevalence in *Ixodes ricinus* ticks across the two sampled populations, Chornobyl City (CC; blue bars) and the Nuclear Power Plant (NPP; gold bars), along with population measures for co-infection and general positivity rate. The asterisk (*) indicates a *Z*-score > 2 and a significant difference at *P* < 0.05

The general *Anaplasmataceae* PCR yielded positive results for 25 of the 106 ticks. Sequencing matched to *A. phagocytophilum* (*n* = 15)*, Anaplasma platys* (*n* = 6) and *Neoehrlichia mikurensis* (*n* = 4) with a percent identity > 97% (> 99% on average) and high query coverage (Fig. 2; Additional file 1: Table S1). As there was a high percent identity in the sequenced region for both *A. platys* and *A. phagocytophilum,* we corroborated the *Anaplasma* sequencing results with a species-specific qPCR, ApMSP2, which does not amplify *A. platys *in silico*.* Tick samples positive for *N. mikurensis* were distributed evenly across the two populations, but we found that four of the six *A. platys*-positive ticks were sampled at CC.

The *F. tularensis* PCR yielded one positive amplicon in a *D. reticulatus* specimen from the NPP, which sequencing identified as a *Francisella*-like endosymbiont of *D. reticulatus* with 99% sequence identity. No other ticks were positive for *F. tularensis* by the PCR.

### qPCR detection of pathogens

A total of 33 *I. ricinus* ticks were positive for *A. phagocytophilum* for both the ‘head’ and ‘body’ extracts, with a higher incidence of positive ticks at the NPP (44.0%; *n* = 22) than at CC (23.1%; *n* = 11; Fig. 2; Additional file 1: Table S1). For this assay, five additional ticks, including one *D. reticulatus* sample, were positive only when the ‘body’ was considered; the ‘head’ extract did not yield any amplification. Thirty-one *I. ricinus* ticks were positive for *B. burgdorferi*, of which 21 (34.0%) were from the NPP and 10 were from CC (11.5%). Three additional ticks were only positive for the ‘body’ extract and did not generate an amplicon for the ‘head’ of the respective tick. For the genospecies assessment, amplicon sequences of 24 of these 31 positive ticks (‘head’ and ‘body’) provided support for *B. burgdorferi* genospecies identification with > 95% sequence identity. The positive samples from CC were identified as *Borrelia afzelii* (*n* = 6), *Borrelia garinii* (*n* = 1), and *Borrelia valaisiana* (*n* = 1) with > 99% sequence identity. The predominant species in NPP samples was *B. afzelii* (*n* = 16). One *I. ricinus* tick from CC (1.9%) and all four sampled *D. reticulatus* ticks from the NPP were positive for *R. raoultii*. The *Bartonella* assay did not yield positive results for any tick extract. For *Babesia* spp., a total of nine *I. ricinus* ticks were positive at similar proportions across the two populations (NPP 8.0%; CC 9.6%). Sequencing from the general *Babesia* spp. qPCR indicated that the eight positives matched most closely to *B. canis*-like with > 97% sequence identity and that one matched most closely to *B. caballi*-like with > 97% sequence identity. The piroplasmid qPCR generated amplicons for four of the eight samples that previously matched closest to *B. canis*. Melt curve analysis of the products indicated that each product had the expected melting temperature for *B. canis* (80.5 °C). Sequencing results from the piroplasmid PCR indicated that the previous *B. canis* positives matched again to *B. canis* with > 99% identity.

Overall tick positivity was also higher at the NPP than at CC, where 62% and 51.8% of ticks were positive for any of the assessed pathogens, respectively. The proportion of ticks from the NPP and CC that were co-infected with two pathogens was 34% and 9.6%, respectively, including one NPP tick for which three different pathogens were detected.

### Pathogen ddPCR assay for dogs

**Fig. 3:**
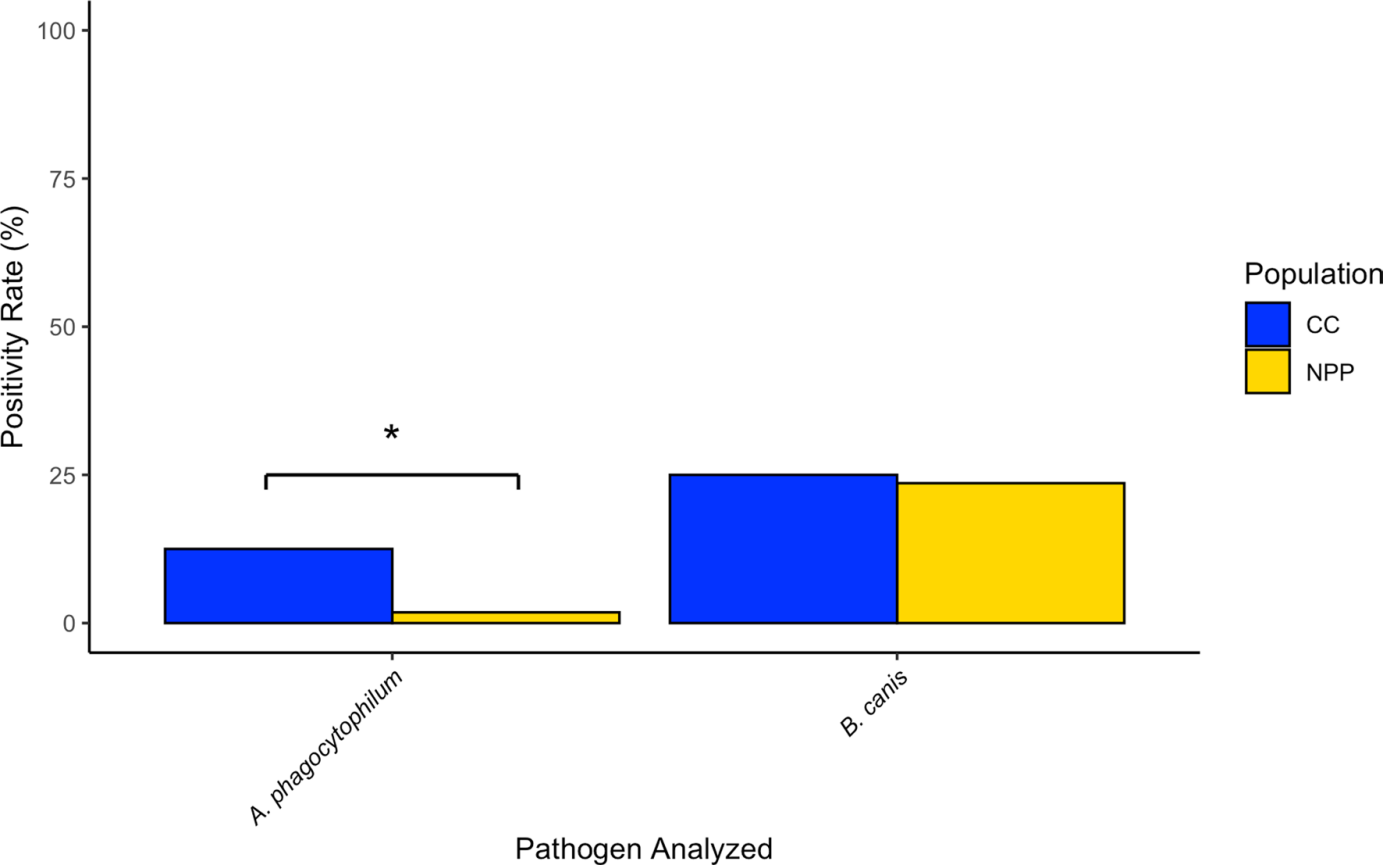
Pathogen prevalence in dogs from Chornobyl City (CC; blue bars) and the Nuclear Power Plant (NPP; gold bars). The asterisk (*) indicates a *Z*-score > 2 and a significant difference at *P* < 0.05

When assessed by ddPCR for the presence of a canine autosomal marker, all dog samples had positive droplets, proving competency of the DNA samples to amplify and thus allowing us to move forward with the pathogen assay. We detected *A. phagocytophilum* in eight of our 111 (7.2%) unique dogs via ddPCR. Seven of the positive dogs were sampled in CC, and one NPP dog had positive droplets. These data indicate that 1.8% of the NPP dogs and 11.7% of the CC dogs were positive for *A. phagocytophilum* (Fig. 3). We noted no persistence for this pathogen’s presence in individuals sampled in both 2018–2019. For *B. canis*, we found that 24.3% (27/111) of the dogs were positive and these were evenly distributed across the sample populations, with 14 of the positive dogs from the CC population. When we considered the dogs sampled in 2018 and 2019, we did find that both CC dogs positive for *B. canis* in 2018 were positive for both years, but of the seven NPP dogs that were positive in 2018, only one retained positivity from 2018 to 2019. Two CC individuals were positive for both *A. phagocytophilum* and *B. canis*, but we found no other overlap in positivity.

**Fig. 4:**
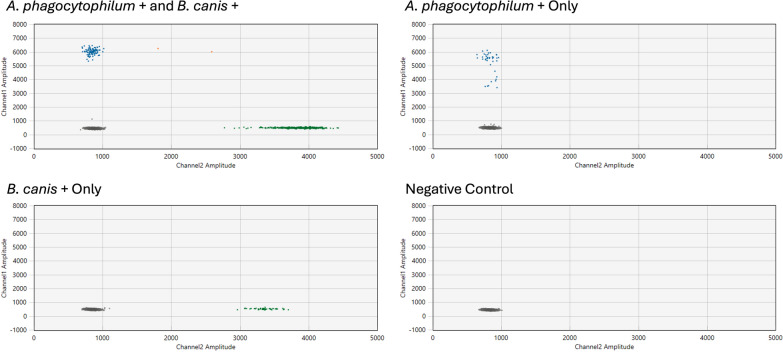
Droplet digital PCR (ddPCR) amplitude plots with selected samples. From left to right, the top row contains a positive control containing a gBlock fragment synthetic control for both *Anaplasma phagocytophilum* and *Babesia canis* (left) and a dog that had positive droplets for *A. phagocytophilum* (right). The bottom row contains a dog with positive droplets for *B. canis* only (left) and a dog negative for both pathogens (right). Fluorescein amidites (FAM; *A. phagocytophilum*) fluorescence is measured on channel 1, and hexachlorofluorescein (HEX; *B. canis*) fluorescence is measured on channel 2

We validated the lower positivity samples by assessment of 100 unaffected dog DNA samples, which yielded only one positive droplet out of over 2,000,000 droplets for each of the *B. canis* and *A. phagocytophilum* probes. This set the background positive droplet rate for each ddPCR assay at < 0.00005%. In addition, the single positive droplets for each assay within the negative control samples did not reach the fluorescence level of droplets in a positive sample (amplitude of approx. 6000 for *A. phagocytophilum*, approx. 3500 for *B. canis*; see Fig. 4) and was therefore not consistent with the amplitude or position of droplets from known positive samples.

### Statistical analysis

We calculated the *Z*-score for each pathogen, to determine differences in pathogen prevalence between the two populations. For ticks, the NPP population had a significantly higher proportion of positivity for *A. phagocytophilum* and *B. burgdorferi* than the CC population, with *Z*-scores of 2.26 and 2.50, respectively (*P* = 0.012; *P* = 0.0062). For the other pathogens, the differences did not reach significance. The difference in proportion of co-infected ticks was significantly higher for the NPP population, with a *Z*-score of 3.07 (*P* = 0.00107). Overall pathogen positivity levels, however, were not significantly different (*Z* = 1.03; *P* = 0.15).

When the dogs were considered, we found that the CC population had a significantly higher proportion of individuals that were positive for *A. phagocytophilum*, with a *Z*-score of 2.23 (*P* = 0.013). There was no significant difference in *B. canis* presence between the NPP and CC populations. We additionally calculated the *Z*-score for persistence of *B. canis*, and found that the difference in *B. canis* retention between the populations was significant (*Z* = 2.27; *P* = 0.012).

## Discussion

In this work, we investigated the potential impact of contrasting types and relative levels of environmental contamination on vectors and vector-borne pathogens at two locations within the CEZ. This study is the first to examine tick pathogens sampled at the NPP and within CC. Furthermore, no previous studies evaluated pathogen prevalence in both ticks and dogs in the CEZ. Pathogen prevalence was higher in ticks at the NPP than in ticks recovered from CC, and the relative proportion of certain pathogens also differed significantly between the two locations. Comparative analysis of the dogs at the two sites also revealed differential pathogen positivity for *A. phagocytophilum*, but, in contrast to the findings with the ticks, the dog population at CC had a higher proportion of positivity. The findings from this study highlight differential pathogen positivity rates in both tick and dog populations sampled at the NPP and in CC, which supports our initial hypothesis.

**Fig. 5:**
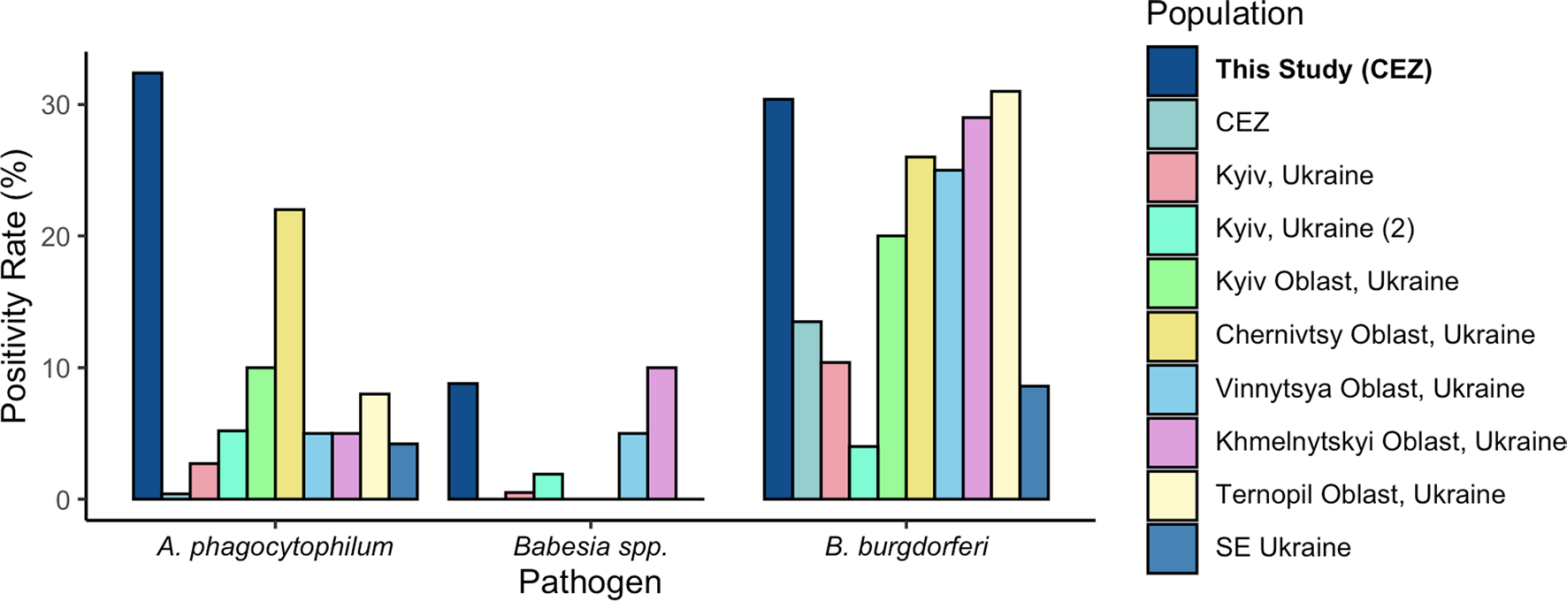
Comparison of pathogen findings in *Ixodes ricinus* ticks for this study (blue) to other studies in Ukraine for three pathogens of interest. *Babesia* spp. not tested for South-Eastern Ukraine. See Table 2 for more detailed summary of these studies

In terms of pathogen positivity overall, 56.8% of our 102 sampled *I. ricinus* ticks were positive for at least one of the eight pathogens assessed. This level of infection is markedly higher than the 11% positivity rate reported previously in almost 700 *I. ricinus* ticks sampled in Kyiv, Ukraine [45]. The positivity rate we identified in the present study is, however, more similar to that found in ticks collected from pet dogs and cats in Southwest Poland, where 65.4% of the *I. ricinus* ticks were positive for pathogens [46]. Comparing pathogen-specific positivity, we note an increased pathogen presence for *A. phagocytophilum* in the *I. ricinus* ticks sampled for our study when compared to the results of other studies in Ukraine (Table 2; Fig. 5). This level is also markedly higher than those found in regions of Poland, which ranged from 1.1% to 21.3% positivity [47–49]. Surprisingly, we did not detect *Bartonella* spp., despite previous reports of *Bartonella* spp. in *I. ricinus* in the CEZ [18]. Pathogen levels of *B. burgdorferi* and *Babesia* spp. were higher than those sampled at sites in close proximity to the CEZ, but these levels are more congruent when compared to studies conducted in more distant oblasts (administrative divisions) of Ukraine and, for *Babesia*, in Southwest Poland [46, 50]. The authors of other studies hypothesized higher than expected pathogen levels for ticks within the CEZ, supporting our findings for *A. phagocytophilum* and *B. burgdorferi* [16, 18]. These two zoonotic pathogens cause two notable emerging tick-borne diseases in Europe: granulocytic anaplasmosis and Lyme disease [51–53].

**Table 2:** Summary of findings for three tick-borne pathogens of interest identified in *Ixodes ricinus* ticks sampled in the Ukraine

Location	*Anaplasma phagocytophilum*	*Borrelia burgdorferi*	*Babesia* spp.	Molecular method	No. ticks analyzed	Collection method	Reference
CEZ (NPP and CC)	32.4%	30.4%	8.8%^x^	qPCR	106	From dogs	This study
CEZ (CC)	0.4%	13.5%	0%	PCR	260	Questing	Rogovskyy et al. [18]
Kyiv, Ukraine	2.7%	10.4%	0.5%	PCR	182	Questing	Rogovskyy et al. [54]
Kyiv, Ukraine (2)	5.2%	4.0%	1.9%^y^	PCR	696	Questing	Didyk et al. [45]
Kyiv Oblast, Ukraine	10%	20%	0%	PCR	20	Questing and animals	Levytska et al. [50]
Chernivtsy Oblast, Ukraine	22%	26%	0%	PCR	23	Questing and animals	Levytska et al. [50]
Vinnytsya Oblast, Ukraine	5%	25%	5%	PCR	20	Questing and animals	Levytska et al. [50]
Khmelnytskyi Oblast, Ukraine	5%	29%	10%	PCR	21	Questing and animals	Levytska et al. [50]
Ternopil Oblast, Ukraine	8%	31%	0%	PCR	13	Questing and animals	Levytska et al. [50]
South-Eastern Ukraine (Zaporizhzhya Oblast)	4.2–7.7%	8.6–12.7%	NT^a^	qPCR	452 (pooled)	Questing and animals/humans	Kovryha et al. [55]

For our study, results are reported across both sample locations within the Chornobyl Exclusion Zone

*CC* Chornobyl City,* CEZ *Chornobyl Exclusion Zone,* NPP* Nuclear Power Plant, qPCR quantitative PCR

^a^NT indicates that pathogen presence was not investigated; for species level identification of *Babeisa *spp., ^x^ corresponds to *Babesia canis*/*B. caballi*, ^y ^corresponds to *B. microti*

Our study reports similar rates of infection by *Babesia* spp*.* and general *Anaplasmataceae* spp*.* (including *A. platys* and *N. mikurensis*) at the two sample locations. Interestingly, all but one of the *Babesia* amplicons closely matched *B. canis*. Vector competency for the transmission of *B. canis* in *I. ricinus* has not been confirmed, despite the presence of the piroplasm in *I. ricinus* in other studies [56, 57]. Therefore, our results cannot be directly linked to transmissibility from tick to host. This species of *Babesia* is typically transmitted by the ornate dog tick, *D. reticulatus*, of which we found very few samples [56, 58].

Of 106 collected ticks, only four were *D. reticulatus*, while the remaining 102 were *I. ricinus*. Previous dragging surveys for questing ticks around the CEZ noted a higher prevalence of *D. reticulatus* [16, 59]. As the sampled ticks were actively feeding on hosts, this may have impacted the species collected and is therefore not representative of all questing ticks that inhabit the CEZ. Also, it is important to note that since the ticks assessed in this study were feeding, pathogen presence may also be affected by the tick’s blood meal. Comparisons between the tick ‘body’ positivity and tick ‘head’ positivity suggest that there are instances where the blood meal was positive for pathogen but that the tick may not be actively transmitting.

In addition to the higher pathogen rates for *A. phagocytophilum* and *B. burgdorferi* between our study sites and other sample locations in Ukraine, we found differential proportions of ticks positive for these pathogens at the two sample sites. There were significantly higher rates of infections in ticks at the NPP than in ticks at CC, and the prevalence rates for the NPP (*A. phagocytophilum*: 44%; *B. burgdorferi*: 42%) were markedly higher than those reported in tick studies conducted in other parts of Ukraine (Table 2). Our findings also indicate an increased overall level of pathogen prevalence in the ticks at the NPP, along with a significantly higher proportion of co-infection with two or more pathogens. As the pool of assessed ticks did not contain any larval or nymphal ticks, the differences in pathogens between the two populations may be due to other environmental or ecological causes. Some studies suggest that certain mammal species, both small [14] and large [7, 15], have rebounded in this area following the 1986 disaster. Additional information on population densities and pathogen prevalence in each pathogen’s respective reservoir species, such as red deer, small rodent species and red foxes, would provide more contextual evidence for these findings and allow for a better assessment of the sylvatic interactions. The area around the NPP is more heavily forested than that around CC, which may facilitate an increase in sylvatic transmission and a higher pathogen positivity rate for the tick population [60]. The most prevalent *B. burgdorferi* genospecies in both populations of ticks was *B. afzelii*, which may be associated with a higher prevalence of pathogen in rodent species; however, further study is required for any conclusion to be drawn [61]. With small rodent species playing a role in the tick-borne pathogen cycle as both reservoir species and early hosts for maturing ticks, it is critical to study the pathogen prevalence in these populations. The radiation releases and widespread destruction of flora and fauna, as well as the subsequent cleanup, likely impacted the populations of small invertebrates, such as ticks, along with the small rodent hosts, which may have affected pathogen spread in the vicinity of the destroyed reactor [62].

**Fig. 6:**
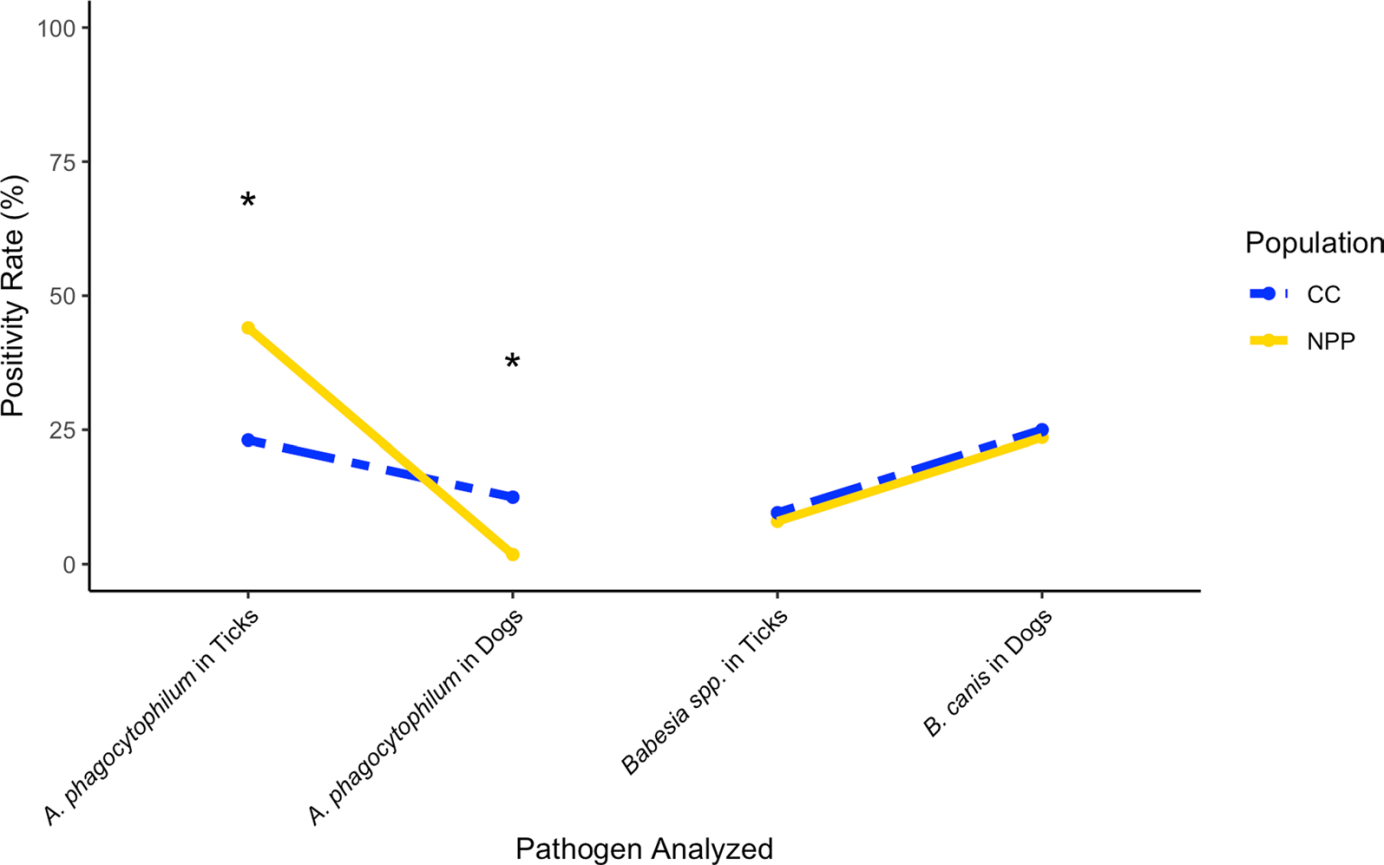
Comparative view of positivity for *Anaplasma phagocytophilum* and *Babesia* spp./*B. canis* in both ticks and dogs. Asterisk (*) indicates *Z*-score > 2 and *P*-value < 0.05

In contrast to pathogen prevalence within the ticks themselves, we found that both of the dog populations sampled around Chornobyl had a higher prevalence of *B. canis* but contradictory levels of *A. phagocytophilum*. The proportion of dogs positive for *B. canis* was higher than the proportion of positive ticks, although this difference may be linked to the possibility of vertical transmission of *B. canis* in dogs [63, 64]. Our data also indicate corresponding positivity levels between *Babesia* spp*.* in ticks and *B. canis* in dogs for both sample locations (Fig. 6). In contrast, dog positivity for *A. phagocytophilum* in both populations was found to be inversely related to tick positivity. The dogs from the CC population had a significantly higher proportion of positivity than those from the NPP population, whereas the ticks at CC had a significantly lower pathogen prevalence than those at the NPP. The persistence of *B. canis* in the dog populations sampled in both 2018 and 2019 also differed. While the sample numbers are small and unevenly distributed between the populations, it is notable that both of the dogs in the CC population which tested positive for *B. canis* in 2018 also tested positive in 2019. Of the seven dogs in the NPP population that tested positive for *B. canis* in 2018, however, only one still tested positive the following year. The reasons for the difference are unclear, and larger sample sizes are needed to validate this observation. It is important to also note that *B. burgdorferi* prevalence could not be compared between the two dog populations despite differential prevalence in the tick populations, as this pathogen localizes in tissues and organs and could not be detected in the dog blood samples analyzed.

Overall, it is notable that significantly different pathogen levels were detected in the two geographically close sample sites (CC and NPP) within the CEZ and that despite the high levels of *A. phagocytophilum* in the tick populations, we failed to detect a similarly high pathogen load in the dogs. This work also highlights interesting trends regarding the persistence of *B. canis* from year to year and the contrasting levels of *A. phagocytophilum* between dog and tick populations. There are a several possible explanations for this reduced *A. phagocytophilum* presence in the dogs at the NPP despite the high level of tick positivity. Possible explanations include differences in the reservoir species, in the vectors or in the dogs themselves, all of which could impact pathogen transmission from ticks to the dogs. Differential tick density in the sampling years could contribute to this difference, but we were unable to assess tick density with dragging surveys for the sampling years and thus are limited in drawing definitive conclusions. Additionally, although all dogs in the present study were unowned and free-breeding, anecdotal evidence suggests that a larger proportion of dogs at the NPP may be given regular doses of tick-preventatives than dogs in CC. This could explain the lower proportion of *A. phagocytophilum* in the dog population, but it does not seem to differentially alter the distribution of *B. canis* between the two populations.

Differing conditions surrounding both these study sites may also help to explain the differences detected between the sample locations. Compared to CC, the NPP site was more highly contaminated by radioactive isotopes, heavy metals and other toxic compounds[65]. Ionizing radiation exposure can diminish microbiome diversity as well as influence pathogen and vector dynamics, which could have contributed to a higher tick pathogen load (reviewed in [28, 66]). Ionizing radiation exposure, along with the other hazardous contaminants present, may impact the immune and inflammatory responses, which, in turn, could influence host response to ticks and tick-borne pathogens [67, 68]. Our earlier published studies suggest directional selection between the dog populations at the NPP and CC sites in genomic regions containing immune regulatory genes. If validated, this observation may reflect the animals’ response to the harsh environmental stressors, and their selective responses could therefore influence susceptibility to infection or persistence of tick-borne pathogens.

## Conclusions

In this study we found high levels of pathogens in feeding ticks collected from the NPP and CC, the latter some 16 km away. A significantly higher prevalence of *A. phagocytophilum* and *B. burgdorferi* were found in ticks at the NPP compared to the CC site, which may reflect very different levels of environmental contamination. In contrast to the ticks themselves, the dogs sampled at the NPP had lower levels of *A. phagocytophilum* compared to those from CC, suggesting that the higher level of toxic materials at the NPP may have affected the dogs’ response to these pathogens or their vectors. Vector-borne pathogens pose serious health risks to both susceptible animal species and the human populations that come into contact with these animals and vectors. Continued study of the tick-borne pathogens and other micro-organisms and parasites, as well as the ways in which animal populations may have adapted to different pathogen prevalence, will add to our understanding of the effects of chronic exposure to radiation and/or toxic chemical exposures and better prepare medical and public health professionals for future environmental disasters.

## Supplementary Information

**Additional file 1.** **Table S1**. Assay results for each tick, including results for both the 'body' compared to the respective 'head' forpositive 'body' samples.

## Data Availability

The data used and/or analyzed during the current study are available from the corresponding author on reasonable request.

## Abbreviations

CC: Chornobyl city
CEZ: Chornobyl Exclusion Zone
ddPCR: Droplet digital PCR
IPC: Internal positive control
NCSU GSL: North Carolina State University Genomic Sciences Laboratory
NPP: Nuclear power plant
qPCR: Quantitative PCR
VBDDL: Vector Borne Disease Diagnostics Laboratory
